# Protein Tyrosine Phosphatase Receptor Type Z Negatively Regulates Oligodendrocyte Differentiation and Myelination

**DOI:** 10.1371/journal.pone.0048797

**Published:** 2012-11-07

**Authors:** Kazuya Kuboyama, Akihiro Fujikawa, Makoto Masumura, Ryoko Suzuki, Masahito Matsumoto, Masaharu Noda

**Affiliations:** 1 Division of Molecular Neurobiology, National Institute for Basic Biology, Aichi, Japan; 2 School of Life Science, The Graduate University for Advanced Studies, 5-1 Higashiyama, Myodaiji-cho, Okazaki, Aichi, Japan; 3 Faculty of Pharmacology II, Asubio Pharma Co. Ltd., 6-4-3 Minatojima-Minamimachi, Chuo-ku, Kobe, Hyogo, Japan; University of Muenster, Germany

## Abstract

**Background:**

Fyn tyrosine kinase-mediated down-regulation of Rho activity through activation of p190RhoGAP is crucial for oligodendrocyte differentiation and myelination. Therefore, the loss of function of its counterpart protein tyrosine phosphatase (PTP) may enhance myelination during development and remyelination in demyelinating diseases. To test this hypothesis, we investigated whether Ptprz, a receptor-like PTP (RPTP) expressed abuntantly in oligodendrocyte lineage cells, is involved in this process, because we recently revealed that p190RhoGAP is a physiological substrate for Ptprz.

**Methodology/Principal Findings:**

We found an early onset of the expression of myelin basic protein (MBP), a major protein of the myelin sheath, and early initiation of myelination *in vivo* during development of the *Ptprz*-deficient mouse, as compared with the wild-type. In addition, oligodendrocytes appeared earlier in primary cultures from *Ptprz*-deficient mice than wild-type mice. Furthermore, adult *Ptprz*-deficient mice were less susceptible to experimental autoimmune encephalomyelitis (EAE) induced by active immunization with myelin/oligodendrocyte glycoprotein (MOG) peptide than were wild-type mice. After EAE was induced, the tyrosine phosphorylation of p190RhoGAP increased significantly, and the EAE-induced loss of MBP was markedly suppressed in the white matter of the spinal cord in *Ptprz*-deficient mice. Here, the number of T-cells and macrophages/microglia infiltrating into the spinal cord did not differ between the two genotypes after MOG immunization. All these findings strongly support the validity of our hypothesis.

**Conclusions/Significance:**

Ptprz plays a negative role in oligodendrocyte differentiation in early central nervous system (CNS) development and remyelination in demyelinating CNS diseases, through the dephosphorylation of substrates such as p190RhoGAP.

## Introduction

**Figure 1:**
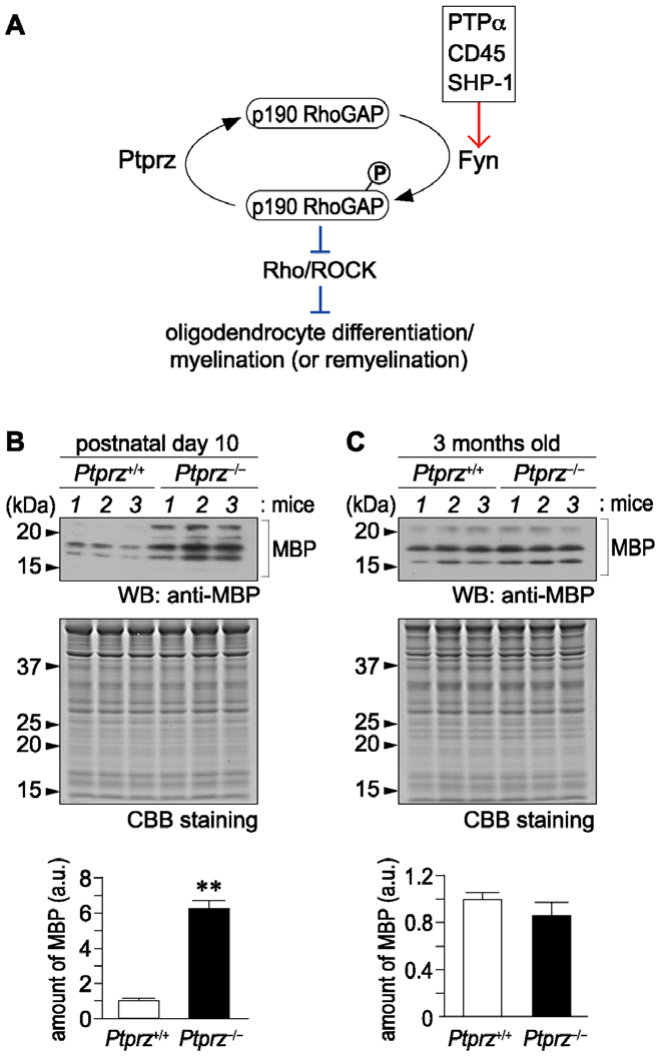
Early onset of MBP expression in the brain of *Ptprz*-deficient mice. ***A***, Schematic drawing of postulated signaling mechanisms of Ptprz and Fyn in oligodendrocyte differentiation and myelination. Fyn and Ptprz may also act on yet unidentified substrates other than p190RhoGAP to regulate the differentiation. The red arrow shows activation, whereas the blunt blue arrows represent inhibition. ***B***, ***C***, Western blot analyses of MBP expression in the cerebral cortex of mice at postnatal day 10 (B), and 3 months old (C). Applied protein amounts were verified by Coomassie Brilliant Blue (CBB) staining. The amounts of MBP are presented as densitometric units normalized to the value for respective wild-type controls, and are shown at the lower position of each panel. Data are the mean ± SEM (*n* = 6 for each group). **p<0.01 (Student's *t*-test). a.u., arbitrary unit.

Myelination is an essential feature of the vertebrate nervous system. Deficiencies in myelination during development, or demyelination following injury or in diseases such as multiple sclerosis (MS) lead to neurological disorders [1]. Several lines of evidence indicate that protein tyrosine phosphorylation is a key event in the differentiation of oligodendrocytes and myelin formation [2]. Tyrosine phosphorylation of cellular proteins is controlled by the balance between activities of protein tyrosine kinases (PTKs) and protein tyrosine phosphatases (PTPs). A Src PTK family member, Fyn is considered important for oligodendrocyte differentiation for the following reasons: Fyn transcription is up-regulated upon oligodendrocyte differentiation [3], Fyn activation induces transcriptional activation of myelin basic protein (MBP), a major protein of the myelin sheath [4], and *Fyn*-knockout mice exhibit a 50% decrease in myelination [5]. p190RhoGAP, a GTPase-activating protein (GAP) for Rho GTPase, is a crucial target of oligodendroglial Fyn signaling, which mediates oligodendrocyte differentiation and myelination. p190RhoGAP is tyrosine phosphorylated by Fyn, and thereby activated during differentiation of oligodendrocytes [6]. The activation of p190RhoGAP to suppress Rho GTPase activity thus represents an essential step for oligodendrocyte maturation and myelination [6]. Given these previous findings, loss of function of PTP activity for p190RhoGAP would be expected to enhance oligodendrocyte differentiation and myelination, or remyelination in experimental demyelinating lesions (see Figure 1A).

Investigations of regulated PTP expression during the differentiation of oligodendrocyte progenitor CG4 cells have revealed four major receptor-like PTPs (RPTPs) among the 11 PTPs identified: Ptpra (also refered to as PTPα), Ptprz (PTPζ/RPTPβ), Ptprs (PTPσ), and Ptprg (PTPγ), in which 40% of all clones were accounted for by Ptpra, followed by Ptprz (30% of the total) [7]. Among them, Ptprz is of particular interest for the following reasons. It is abundantly expressed in neuronal and glial cells in the central nervous system (CNS) [8]–[10], and especially abundant in the subventricular zone where the oligodendrocyte progenitors reside in early developmental stages [11]. Ptprz is the most abundant RPTP in A2B5-positive human white matter progenitor cells (WMPCs), and a lentiviral short-hairpin RNA (shRNA) construct for *Ptprz* abolished the expansion of undifferentiated WMPCs and promoted their oligodendrocyte differentiation in a culture system [12], suggesting that Ptprz is a negative regulator for the differentiation. Notably, we have revealed that Ptrpz has multiple physiological substrates including p190RhoGAP [13]–[18].

Although these findings strongly suggested that loss of Ptprz function leads to enhancement of the tyrosine phosphorylation of p190RhoGAP, and thereby enhances oligodendrocyte differentiation and myelination, the role of Ptprz in the oligodendrocyte lineage remains controversial. So far, three lines of knockout mice lacking *Ptprz* have been generated with different strategies by three independent groups including ours [8], [9], [19], and all three lines appear grossly normal. Among them, the knockout line reported by Harroch *et al.* exhibits a fragility of myelin in the CNS [19] and impaired remyelination after experimental autoimmune encephalomyelitis (EAE)-induced demyelination [20], suggesting conversely a positive role for Ptprz in oligodendrocyte survival and in recovery from demyelinating disease. However, both Harroch *et al*. [19] and Lafont *et al*. [9] reported no changes in the conduction velocity of the central nerve in their knockout mice.

In the present study, we therefore examined the roles of Ptprz in oligodendrocyte differentiation and myelination during development, and remyelination in experimental demyelinating diseases, by using knockout mice generated in our laboratory [8]. We found an early onset of the expression of MBP and early initiation of myelination during development of the brain in *Ptprz*-deficient mice. In addition, adult *Ptprz*-deficient mice exhibited a lower susceptibility to EAE than wild-type mice.

## Results

**Figure 2:**
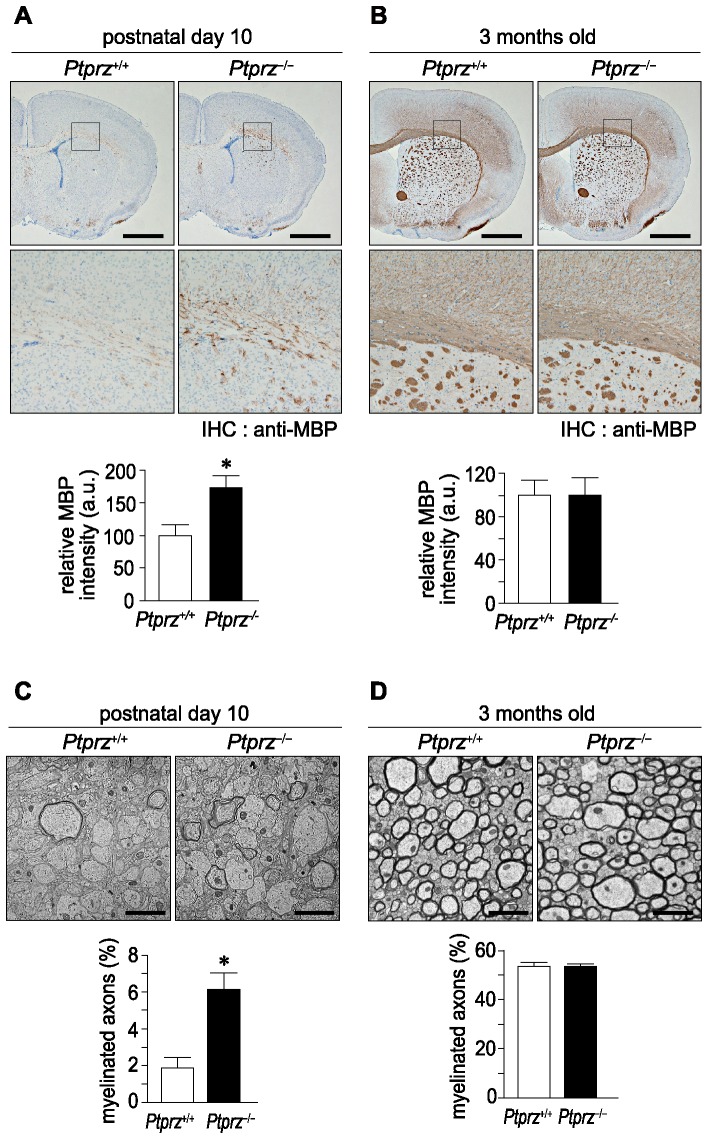
Early initiation of myelination in *Ptprz*-deficient mice. ***A***, ***B***, Immunohistochemical analyses of MBP expression in mouse brains at postnatal day 10 (A), and 3 months old (B). Scale bars, 1 mm. The results of the densitometric analysis of MBP signals are normalized to the value for respective wild-type controls, and shown at the lower position of each panel. Data are the mean ± SEM (*n* = 3 for each group). *p<0.05 (Student's *t*-test). a.u., arbitrary unit. ***C***, ***D***, Electron micrographs of transverse sections at the corpus callosum from mice at postnatal day 10 (C), and 3 months old (D). Scale bars, 2 µm. Percentages of myelinated axons in total axons are shown at the lower position of each panel. Data are the mean ± SEM (*n* = 4 for each group). *p<0.05 (Student's *t*-test).

When MBP expression during development was compared between wild-type and *Ptprz*-deficient mice by Western blotting, the amount of MBP protein in the brain was found to be significantly higher in the *Ptprz*-deficient mice at postnatal day 10 (Figure 1B), but almost the same at 3 months of age (Figure 1C). In addition, immunohistochemical staining of MBP was wide spread and stronger in the corpus callosum of *Ptprz*-deficient mice as compared with that of wild-type mice at postnatal day 10 (Figure 2A), but again no differences were detected between the adult groups (Figure 2B). Consistent with the early expression of MBP, electron microscopy of the corpus callosum revealed that *Ptprz*-deficient mice at postnatal day 10 (Figure 2C), but not at 3 months of age (Figure 2D), had more myelinated axons than the wild-type animals.

**Figure 3:**
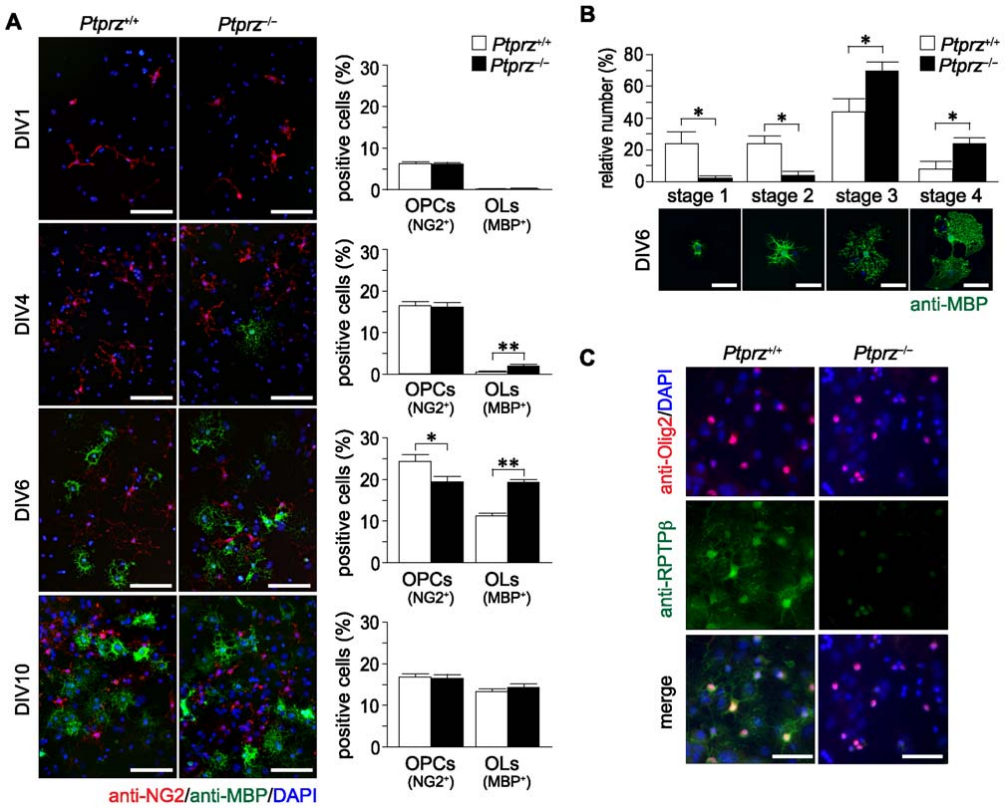
Early onset of oligodendrocyte differentiation in *Ptprz*-deficient mice. ***A***, Immunohistochemistry of cultured oligodendrocyte precursor cells (OPCs, NG2-postive cells, red) and oligodendrocytes (OLs, MBP-postive cells, green) from *Ptprz*-deficient mice and wild-type mice. Scale bars, 100 µm. The percentages of OPCs and OLs among total cells (DAPI-positive nuclei, blue) are shown at the right of each panel. Data are the mean ± SEM from five independent experiments. *p<0.05 and **p<0.01 (Student's *t*-test). ***B***, Morphological assessment of cultured OLs at DIV6. The MBP-postive cells were classified into four categories. Representative images are shown in lower panels; Stage 1, three or less primary processes longer than a cell body with minimal development of secondary and tertiary processes; Stage 2, three or more primary processess with moderate secondary and tertiary processes; Stage 3, five or more primary processes with extensive secondry and teriary processes; Stage 4, extending myelin-like membrane structures and branched processes. Scale bars, 50 µm. Data are the mean ± SEM from three independent experiments. *p<0.05 (Mann-Whitney *U*-test). ***C***, Ptprz expression in oligodendrocyte lineage cells. Cultured cells at DIV10 were triple stained with anti-RPTPβ (specific for Ptprz receptor isoforms, green), anti-Olig2 (red), and DAPI (blue). Scale bars, 50 µm.

We examined the time course of oligodendrocyte differentiation in primary culture of brain cells from newborn mice. As shown in Figure 3A, double immunostaining with anti-NG2 proteoglycan and anti-MBP antibodies showed that the proportion of oligodendrocyte precursor cells (OPCs, NG2-positive cells) was initially similar between the two genotypes at day *in vitro* (DIV) 1, but OPCs from *Ptprz*-deficient mice became significantly fewer at DIV6. Importantly, oligodendrocytes (MBP-positive cells) increased concomitantly. The difference between the two genotypes was most pronounced at DIV6 and disappeared later at DIV10. This suggests that cultured OPCs from *Ptprz*-deficient mice show earlier maturation than those from wild-type mice. Indeed, MBP-positive cells of *Ptprz*-deficient mice showed more differentiated features of oligodendrocytes with highly branched processes and larger membranous expansions at DIV6, as compared with the wild-type control (Figure 3B).

It is known that three splicing variants of Ptprz are expressed from a single gene: Ptprz-A, the full-length receptor form; Ptprz-B, the short receptor form with a deletion in the extracellular region; and Ptprz-S, the secretory variant of Ptprz-A, which is also known as phosphacan/6B4 proteoglycan (6B4PG) (see Figure S1A) [21]. Double staining of the cultured cells with antibodies against oligodendrocyte transcription factor 2 (anti-Olig2, a marker of oligodendrocyte lineage cells) and against the Ptprz receptor isoforms (anti-RPTPβ, see Figure S1A) verified the expression of Ptprz receptors in oligodendrocyte lineage cells (Figure 3C), as was reported previously [9], [10], [12]. Taken altogether, it is considered that Ptprz is negatively involved in the control of oligodendrocyte differentiation and myelination of CNS axons during development.

Prior to the animal study of EAE, we determined the expression profile of Ptprz in the spinal cord of wild-type mice. Western blotting revealed the presence of the full-length core proteins of Ptprz-S and Ptprz-B, and several proteolytic Ptprz fragments in the spinal cord (Figure S1 and Note S1), which is essentially the same as that observed in the brain 22–23. Although the core protein of Ptprz-A was not detected, mRNAs for all the three isofoms were detected by reverse transcription polymerase chain reaction (RT-PCR) analysis (Figure S2A), indicating that almost all Ptprz-A proteins are constitutively processed in the spinal cord as in the brain [21].

**Figure 4:**
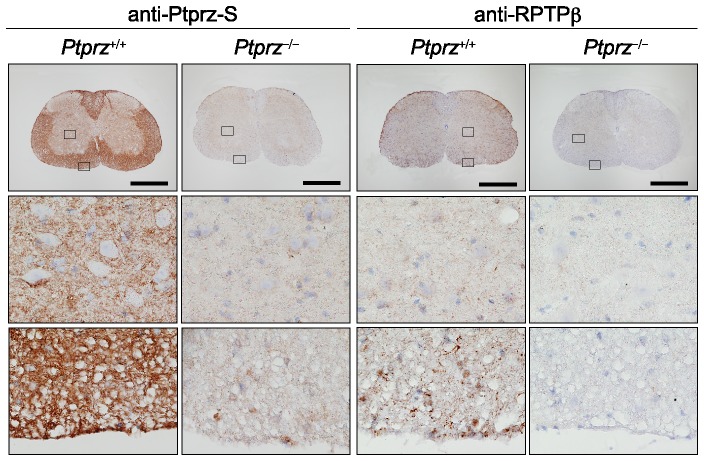
Expression of Ptprz in the spinal cord of adult mice. Immunohistochemical staining of the spinal cord with anti-Ptprz-S (left panels) and anti-RPTPβ (right panels). The lower images are enlargements of the areas enclosed by squares in the upper images. Scale bars, 500 µm.

Immunohistochemical staining with anti-Ptprz-S, which recognizes the extracellular region of all three isoforms (see Figure S1A) showed strong signals diffusely distributed in the white matter, but weak signals were also present in the gray matter, in the spinal cord (Figure 4, left panels). Anti-RPTPβ staining revealed that Ptprz-B and probably C-terminal fragments of the receptor isoforms are located mainly in the white matter, where they showed a punctate staining pattern (Figure 4, right panels). These results indicate that all Ptprz proteins are distributed predominantly in the white matter, where the structures are mainly composed of myelinated axons. No staining was observed with either antibody in the spinal cord of *Ptprz*-deficient mice.

**Figure 5:**
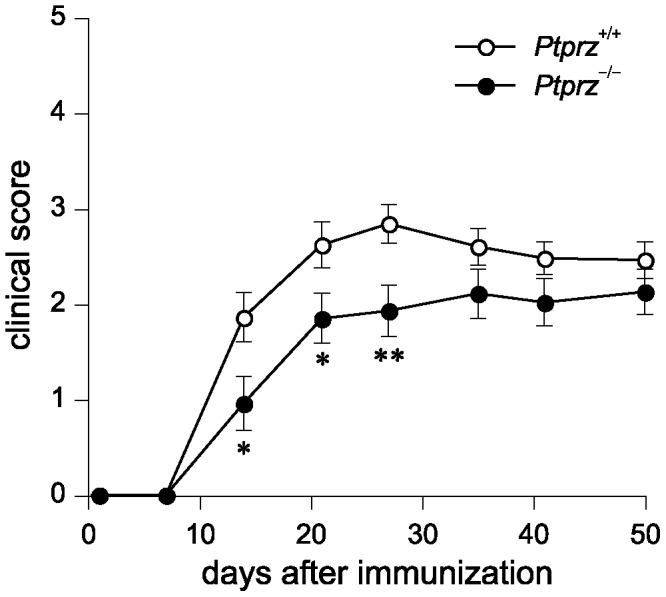
Reduced clinical severity of EAE in *Ptprz*-deficient mice. Clinical scores in wild-type mice and *Ptprz*-deficient mice after the MOG peptide injection. Clinical scores are 0, no disease; 1, limp tail; 2, ataxia and/or paresis of hindlimbs; 3, paralysis of hindlimbs and/or paresis of forelimbs; 4, tetraparalysis; 5, moribund or death. Data are the mean ± SEM (*Ptprz* ^+/+^, *n* = 25; *Ptprz* ^−/−^, *n* = 23). The comparison of clinical scores between the two groups at each time point was performed with Mann-Whitney's *U*-test, *p<0.05, **p<0.01.

Then, we examined the susceptibility of *Ptprz*-deficient mice to myelin/oligodendrocyte glycoprotein (MOG)-induced EAE, a widely-accepted model for studying the clinical and pathological features of MS. Following immunization with the MOG peptide, both *Ptprz*-deficient mice and wild-type mice began to develop EAE with characteristic symptoms on around day 10. However, *Ptprz-*deficient mice showed significantly better clinical scores on days 14, 21 and 28, and the tendency continued until day 50, as compared with wild-type mice (Figure 5). The incidence among wild-type mice reached 100% (*n* = 25/25) by day 14, whereas 17.4% (*n* = 4/23) of *Ptprz*-deficient mice did not show any clinical signs of EAE even at the end of the experiments. The maximum clinical score achieved during 50 days was significantly better in *Ptprz*-deficient mice (2.48±0.26), compared with wild-type mice (3.16±0.12) (*p* = 0.035 by Mann-Whitney *U*-test). Thus, a deficiency of Ptprz reduces the severity, but does not delay the onset, of EAE.

**Figure 6:**
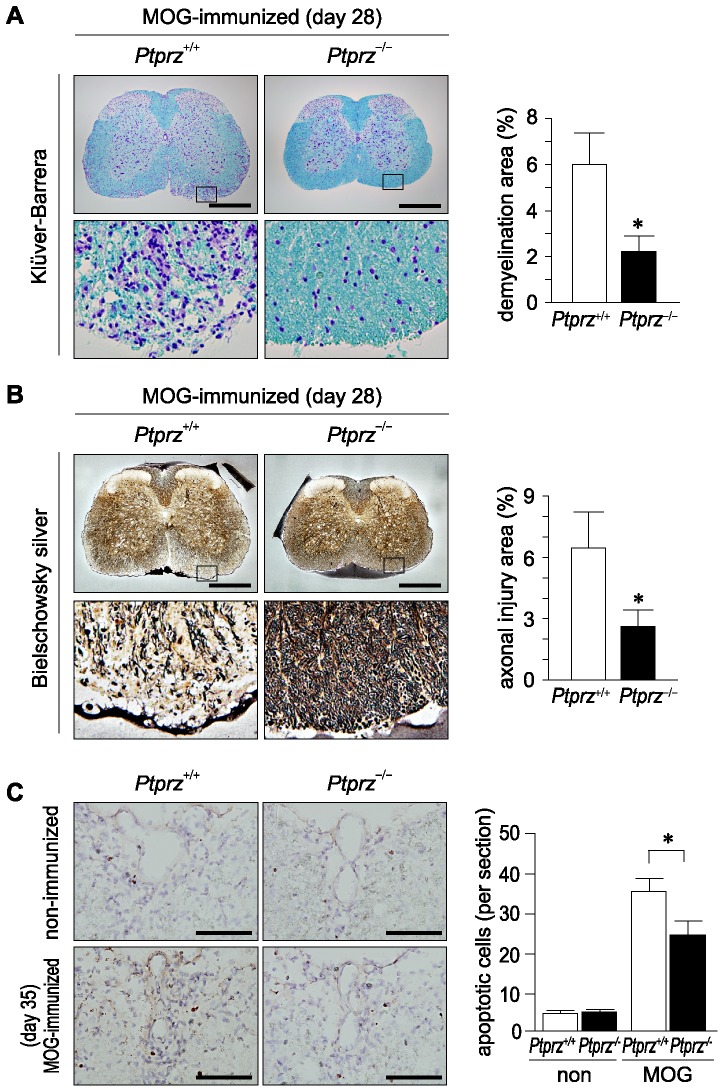
Reduced tissue damage and increased oligodendrocyte survival in *Ptprz*-deficient mice with EAE. ***A***, ***B***, Klüver-Barrera (A) and Bielschowsky silver staining (B) of the spinal cord obtained from wild-type and *Ptprz*-deficient mice 28 days after MOG immunization. The lower images are enlargements of the areas enclosed by squares in the upper images, respectively. The extent of demyelination and axon injury determined by Klüver-Barrera staining and Bielschowsky silver staining is shown as the percentage of damaged areas at the right of each panel. Scale bars, 500 µm. Data are the mean ± SEM (*n* = 10 for each group). *p<0.05 (Student's *t*-test). ***C***, TUNEL staining of spinal cord sections 35 days after MOG immunization, or non-immunized control animals. Scale bars, 100 µm. TUNEL-positive cells were counted in six sections from each animal, and the numbers of TUNEL-positive cells per section are shown at the right. Data are the mean ± SEM (*n* = 6 for each group). *p<0.05 (Student's *t*-test).

Histological analyses of the spinal cord lesion on day 28, at the peak of EAE clinical severity, revealed the demyelination (Figure 6A) and axonal injury (Figure 6B) to be significantly attenuated in *Ptprz*-deficient mice as compared with wild-type mice, which is consistent with the clinical observation. However, it should be noted that the associated reduction in demyelination and axonal injury of *Ptprz*-deficient mice was still significant on day 50 (Figure S3). To determine whether Ptprz deficiency affects the susceptibility to EAE through the promotion of cell survival, we examined the degree of cell death in the EAE-affected mice. Terminal deoxynucleotidyl transferase-mediated dUTP nick-end labeling (TUNEL) staining showed that apoptotic cell numbers were significantly reduced in the spinal cord of *Ptprz*-deficient mice after EAE induction (day 35; Figure 6C). Double staining of the TUNEL-positive cells together with cell-type markers for oligodendrocyte lineage cells (anti-Olig2), T-cells (anti-CD3), and macrophages/microglia (anti-Iba1) revealed that almost all apoptotic cells were oligodendrocyte lineage cells, and their proportions were similar between the two genotypes (Figure S4).

**Figure 7:**
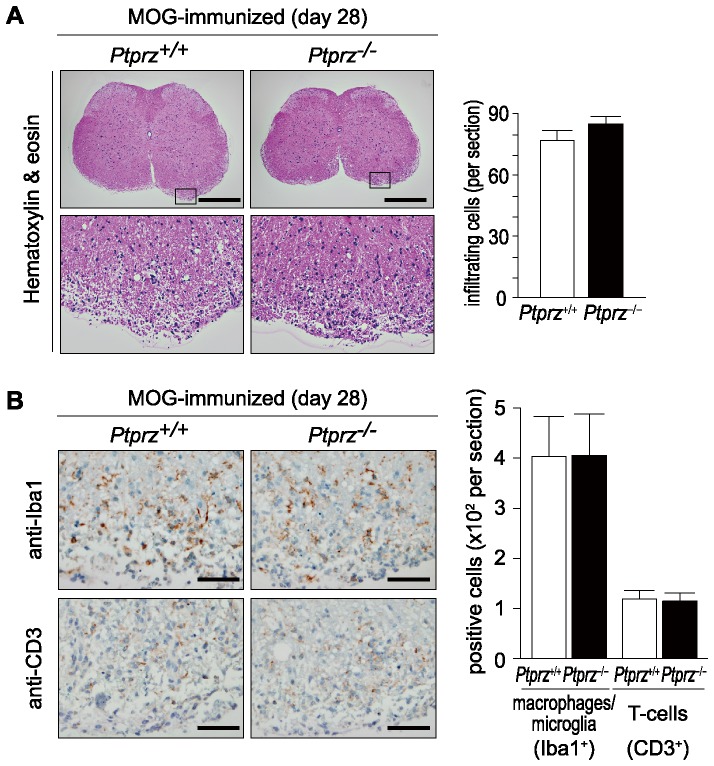
No genotypic differences in infiltrating T-cells and macrophages/microglia within the spinal cord after EAE induction. ***A***, Hematoxylin and eosin staining of the spinal cord 28 days after MOG immunization. The lower images are enlargements of the areas enclosed by squares in the upper images. Scale bars, 500 µm. The numbers of infiltrating cells per section are shown at the right. Data are the mean ± SEM (*n* = 10 for each group). ***B***, Immunohistochemistry of infiltrating T-cells (detected with anti-CD3) or macrophages/microglia (with anti-Iba1) in the spinal cords 28 days after MOG immunization. Scale bars, 50 µm. The numbers of CD3-positive or Iba1-positive cells are shown at the right. Data are the mean ± SEM (*n* = 10 for each group). No significant differences were detected between the two genotypes.

**Figure 8:**
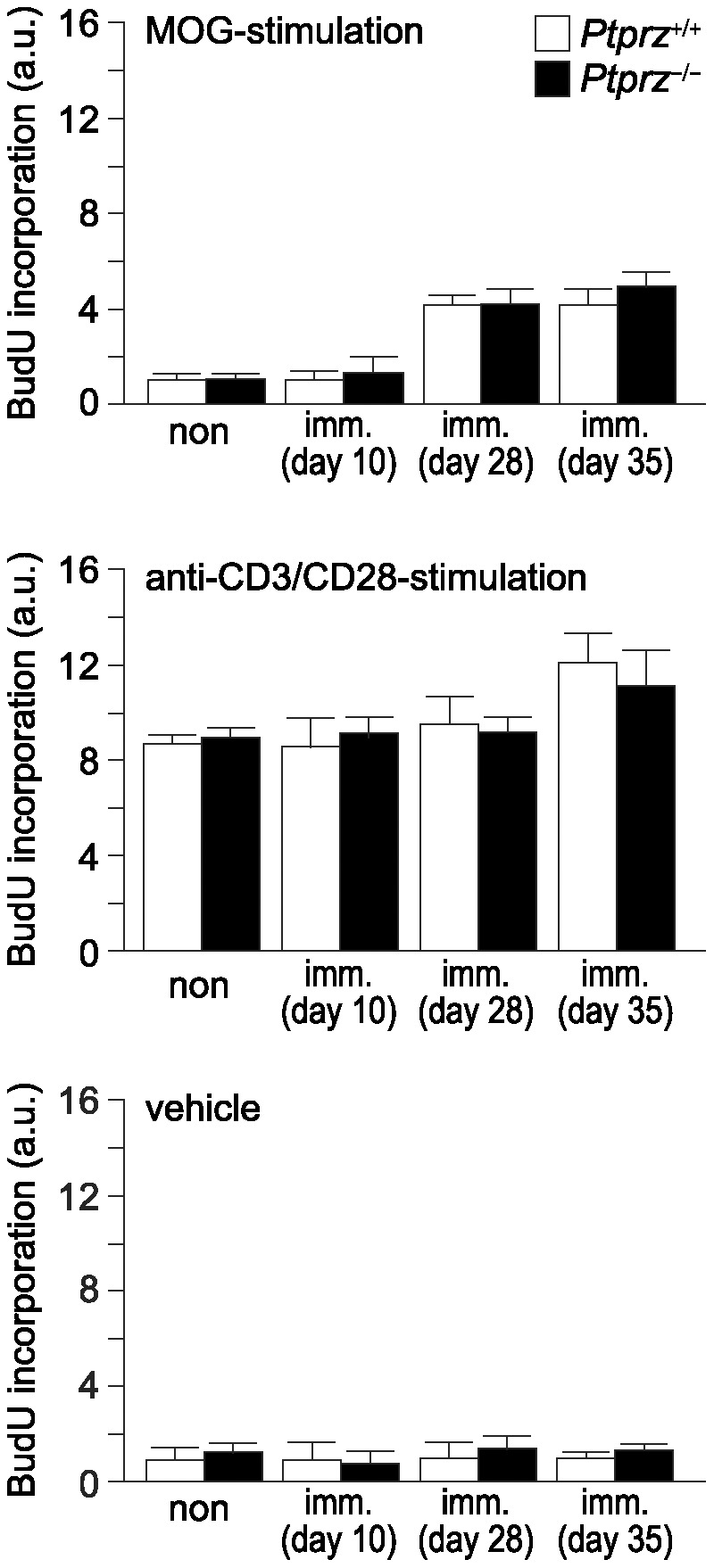
Normal proliferative responses in T-cells of *Ptprz*-deficient mice. T-cell preparations taken from the axillary and inguinal lymph nodes of mice at 10, 28, or 35 days after MOG immunization (imm.), or non-immunized control (non) mice were cultivated in the presence of the MOG peptide (top), anti-CD3/CD28 antibodies (middle), or vehicle (bottom). BrdU incorporation was measured as an index of cell proliferation, and is expressed as the relative change (fold-increase) compared with the vehicle-treated sample of the non-immunized wild-type control. Data are the mean ± SEM (*n* = 6 for each group). No significant differences were detected between the two genotypes. a.u., arbitrary unit.

Despite the reduced tissue damage in *Ptprz*-deficient mice on day 28 and 50 after MOG immunization, there was no siginificant difference in the number of inflammatory cells in hematoxylin and eosin-stained sections between the two genotypes (Figure 7A and Figure S5A). Immunohistochemistry revealed similar numbers of infiltrating T-cells (CD3-positive cells) and macrophages/microglia (Iba1-positive cells) in the spinal cord sections in wild-type and *Ptprz*-deficient mice (Figure 7B and Figure S5B). These results suggest that the reduced tissue damage in *Ptprz*-deficient mice is not attributable to an inhibition of infiltration by inflammatory cells. We verified this view by conducting *in vitro* proliferation assays of T-cells harvested from the axillary and inguinal lymph nodes, obtained from mice after MOG-peptide immunization or without immunization. There were no differences in the T-cell response to the stimualtion with MOG peptide, with anti-CD3/CD28 beads, or with vehicle control between the two genotypes, both under MOG-immunized and non-immunized conditions (Figure 8). Of note, CD3-positive T-cells harvested from the axillary and inguinal lymph nodes were negative for anti-RPTPβ by immunocytochemistry (Data not shown), and for mRNA expression of any Ptprz isoforms by RT-PCR (Figure S2B).

**Figure 9:**
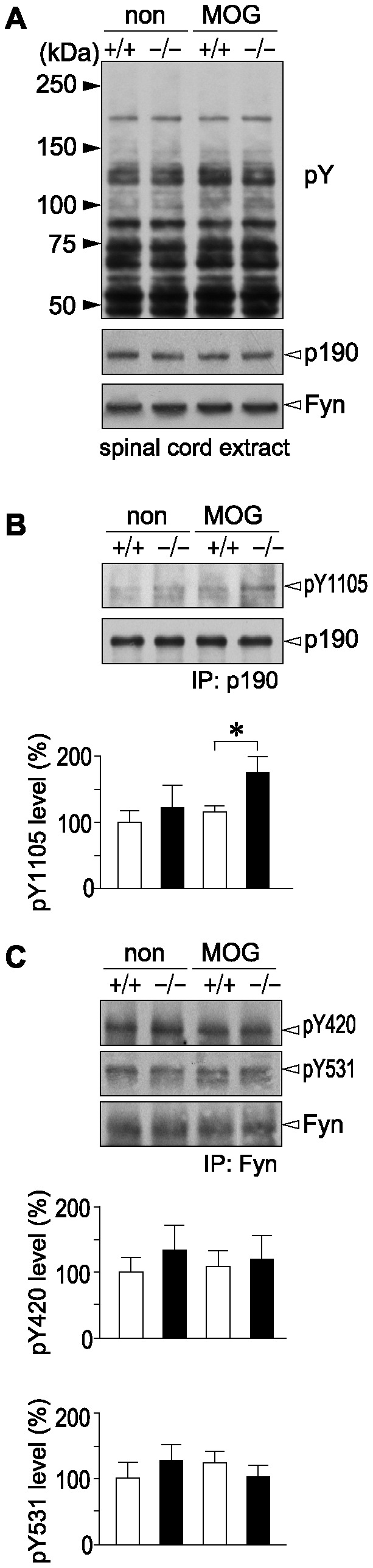
Increased phosphorylation of Tyr 1105 on p190RhoGAP in the spinal cord of *Ptprz*-deficient mice after EAE induction. ***A***, Overall tyrosine phosphorylation patterns of total protein and expression of p190RhoGAP and Fyn in the spinal cord. The third to sixth lumbar spinal cord extracts were prepared from wild-type (+/+) and *Ptprz*-deficient mice (−/−) 35 days after MOG immunization, or non-immunized control animals, and examined by Western blotting using anti-phosphotyrosine PY20 (top), anti-p190RhoGAP (middle), and anti-Fyn (bottom) antibodies, respectively. ***B***, Tyrosine phosphorylation of Tyr 1105 on p190RhoGAP. The spinal cord extracts were immunoprecipitated with anti-p190RhoGAP antibody and immunoblotted with anti-pY1105 p190RhoGAP (upper), or anti-p190RhoGAP (lower). The densitometric data for anti-pY1105 p190RhoGAP signals are presented as a percentage of the non-immunized wild-type control, and shown at the bottom. Data are the mean ± SEM (*n* = 4 pooled samples from two animals per each group). *p<0.05 (Student's *t*-test). ***C***, No siginificant differences in tyrosine phosphorylation of Fyn among the four groups. The spinal cord extracts prepared as above were immunoprecipitated with anti-Fyn antibody and immunoblotted with anti-pY420 (top), anti-pY531 (middle), or anti-Fyn (bottom). The densitometric data for anti-pY420 and anti-pY531 signals are presented as a percentage of the non-immunized wild-type control, and shown at the bottom. Data are the mean ± SEM (*n* = 4 pooled samples from two animals per group). No significant differences were detected between the two genotypes.

It is known that Fyn activation leads to transcriptional activation of MBP [5], and that p190RhoGAP is primarily phosphorylated at Tyr 1105 by Fyn prior to the differentiation of oligodendrocytes [6]. We previously reported that the phosphorylated tyrosine at 1105 of p190RhoGAP is selectively dephosphorylated by Ptprz [14], [15]. To investigate whether the tyrosine phosphorylation of p190RhoGAP is affected differently by EAE in the two genotypes, we examined the phosphorylation of p190RhoGAP by Western blotting in the spinal cord of mice 35 days after MOG immunization, or of non-immunized mice. We could not detect any significant differences in overall protein tyrosine phosphorylation patterns of the spinal cord extracts, and in expression of either p190RhoGAP or Fyn, among the four groups (Figure 9A). However, *Ptprz*-deficient mice with EAE had higher levels of Tyr 1105-phosphorylation in p190RhoGAP than wild-type mice with EAE (Figure 9B). Taken together with the finding that there were no significant differences in the tyrosine phosphorylation of Fyn (Figure 9C), these results strongly support the view that Ptprz functions as the counterpart of Fyn and thereby suppresses oligodendrocyte differentiation and remyelination in EAE.

**Figure 10:**
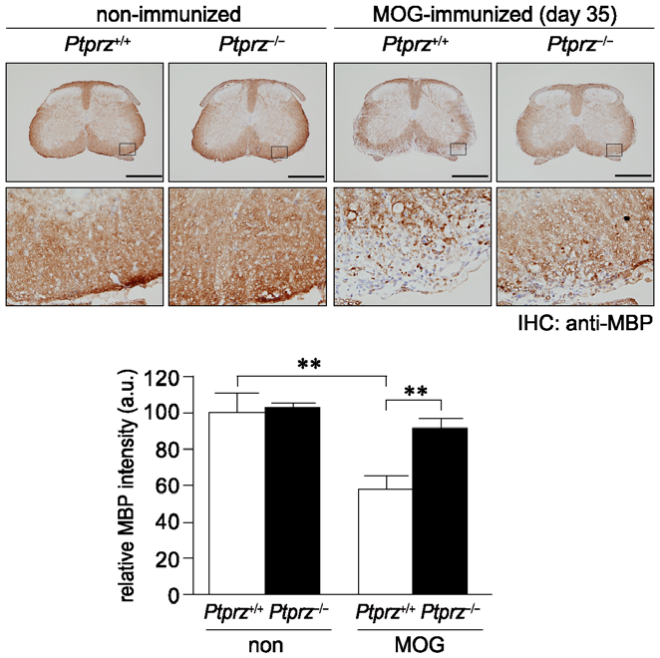
Reduced MBP loss in *Ptprz*-deficient mice after EAE induction. Anti-MBP staining of the spinal cord sections from wild-type and *Ptprz*-deficient mice 35 days after MOG immunization, or non-immunized control mice. The lower images are enlargements of the areas enclosed by squares in the upper images. Scale bars, 500 µm. The densitometric data for MBP signals are expressed as the relative change (fold-increase) compared with the non-immunized wild-type mice, and shown at the bottom. Data are the mean ± SEM (*n* = 6 for each group). **p<0.01 (Student's *t*-test). a.u., arbitrary unit.

To further confirm this notion, we examined whether MBP expression is altered at the protein level. Immunohistochemical analyses revealed that the amount of MBP in the white matter of the spinal cord in wild-type mice was markedly decreased on day 35 after MOG immunization, but EAE-induced loss of MBP in *Ptprz*-deficient mice was significantly suppressed (Figure 10). We detected the difference in the MBP staining between the two genotypes on day 28, while not on day 10, after MOG immunization (Figure S6). Taken altogether, these results support the validity of our hypothesis that Ptprz down-regulates differentiation and remyelination in experimental demyelinating lesions through dephosphorylation of p190RhoGAP.

## Discussion

In this study, we found an early onset of the expression of MBP, a major protein of the myelin sheath, and early initiation of myelination during brain development in *Ptprz*-deficient mice. Also, oligodendrocytes appeared earlier in primary culture of the brain from *Ptprz*-deficient mice than wild-type mice. Adult *Ptprz*-deficient mice showed significant resistance to MOG-induced EAE as compared with wild-type mice. Taken together with the increased tyrosine phosphorylation of p190RhoGAP and reduced MBP loss in *Ptprz*-deficient mice after EAE induction, it became clear that Ptprz acts as a negative regulator for oligodendrocyte differentiation and myelination/remyelination. Selective inhibition of Ptprz signaling therefore could be an effective and plausible therapeutic strategy for treating demyelinating diseases.

A Src PTK family member, Fyn is important for oligodendrocyte differentiation and the transcriptional activation of MBP [2]–[6]: The *Fyn*-knockout mouse exhibits a 50% decrease in myelination [5]. On the other hand, we found that *Ptprz*-deficient mice show an early onset of MBP expression (Figure 1B and 1C). In adult wild-type mice, Ptprz-B is the major isoform in the spinal cord (Figure S1C), as well as in the brain [21] at the protein level. So, this isoform of Ptprz likely acts as the major counterpart of Fyn through the dephosphorylation of its substrates including p190RhoGAP in animals with EAE (see Figure 1A). To our knowledge, PTP molecules other than Ptprz are all positive regulators of oligodendrocyte differentiation and myelination [24]–[27], because dephosphorylation of the inhibitory tyrosine residue in Fyn, C-terminal Tyr 531, by PTPs is an essential event for Fyn activation. PTPα is known as an important positive regulator of Fyn activation, and dephosphorylates Fyn at this tyrosine residue [24]. The activation of Fyn is reduced in cells lacking PTPα, and *PTPα*-deficient mice show defective myelination in the brain [25]. In addition, it has been reported that the knockout of another RPTP, CD45 [26], or a cytosolic PTP, SHP-1 [27], in mice also causes mild dysmyelination, or reduced MBP expression.

p190RhoGAP is tyrosine phosphorylated by Fyn during the differentiation of oligodendrocytes, and the activation of p190RhoGAP thereby represents essential signaling for oligodendrocyte maturation [6]. We previously identified p190RhoGAP as one of the physiological substrates for Ptprz [14], [15]. The tyrosine phosphorylation of p190RhoGAP appears to be controlled by Ptprz also in demyelinating diseases. After EAE induction, the phosphorylation of p190RhoGAP at Tyr 1105 was significantly increased (Figure 9B) and the loss of MBP was markedly suppressed (Figure 10 and Figure S6) in the spinal cord of *Ptprz*-deficient mice, as compared with wild-type mice. It is therefore conceivable that the lack of Ptprz or selective inhibition of its PTP activity in oligodendrocyte lineage cells results in a decreased susceptibility to EAE. Interestingly, such a functional relationship between Fyn and Ptprz has been suggested in another context: *Fyn*-deficient mice show an age-dependent decrease in long-term potentiation (LTP) in the hippocampus [28], while *Ptprz*-deficient mice show an age-dependent enhancement of LTP [29]. Notably, the tyrosine phosphorylation of p190RhoGAP is decreased by learning experiences in the hippocampus of wild-type mice but not of *Ptprz*-deficient mice [15]. It is thus likely that Ptprz acts as a negative regulator of Fyn-p190RhoGAP signaling in several biological processes.

We previously revealed that growth factors such as pleiotrophin/heparin-binding growth-associated molecule (HB-GAM) and midkine bind to the extracellular region of Ptprz [30]–[32]. Pleiotrophin and midkine are the two members of a family of heparin-binding growth factors, which inactivate Ptprz by inducing oligomerization and increase the tyrosine phosphorylation of the substrates of Ptprz [33]. Midkine deficiency reportedly attenuates EAE, due to an expansion of the regulatory T-cell population and a decrease in the number of autoreactive T-helper cells [34]. However, this effect of midkine deficiency on EAE symptoms is thought to reflect the ligand activities for midkine receptors other than Ptprz, such as anaplastic lymphoma kinase (ALK), integrins, or low density lipoprotein receptor-related protein (LRP) [35], because Ptprz expression was not detected in T-cells harvested from the axillary and inguinal lymph nodes (Figure S2B) and the number of inflammatory cells infiltrating the spinal cord was not altered in the MOG-injected *Ptprz*-deficient mice (Figure 7 and Figure S5).

In the normal spinal cord, the secreted Ptprz-S (or Z_A_-ECF) and several extracellular fragments of Ptprz were abundantly present (Figure 4; see also Figure S1), as well as in the brain [21]. The soluble extracellular fragments, Z_A_-ECF and Z_B_-ECF, are generated by tumor necrosis factor-α-converting enzyme (TACE/ADAM-17)-dependent or matrix metalloproteinase-9 (MMP-9)-dependent ectodomain shedding of Ptprz-A and Ptprz-B, respectively [21]. Interestingly, metalloproteinases have also received attention regarding their roles in the pathogenesis of MS [36]: MMP-9 levels are elevated in the brain of EAE mice [37], and *MMP-9*-deficient mice are resistant to EAE development [38]. Here it should be noted that, the extracellular domain of Ptprz binds to various cell adhesion molecules such as Nr-CAM, L1/Ng-CAM, F3/contactin, neural cell adhesion molecule (NCAM), and TAG1/axonin-1, and also extracellular matrix molecules such as tenascin-C and tenascin-R [39]. It has been reported that the binding of the axonal cell adhesion molecule L1 to the oligodendroglial cell surface leads to activation of Fyn and enhances MBP mRNA translation in the cells [40]. Contactin is reportedly one of the oligodendroglial surface receptors for L1 and mediates Fyn activation [41]. Therefore, aberrant regulation of the proteolytic processing of Ptprz may cause an erroneous contactin signaling in oligodendrocyte lineage cells by producing inhibitory ligands.

It has also been shown that the interaction between glial Ptprz and neuronal contactin triggers bidirectional signaling between glial cells and neurons [42]. The stimulation of localized MBP protein synthesis by axon–glial signaling may initiate the formation of myelin at specific time points during development and specific axonal regions depending on the presentation of the appropriate axonal ligands. On the other hand, an *in vitro* finding that recombinant proteins corresponding to N-terminal portions of Ptprz prompt excitotoxic neuronal death [43] suggests that neural tissue damage may be exacerbated by the accumulation of degradated extracellular fragments of Ptprz. Their proper functioning through different receptors must be affected by a deficiency of Ptprz.

The results of the present study are opposite to previous findings by Harroch *et al.* using a mouse line lacking Ptprz, in which they revealed fragility of myelin in the CNS [19] and impaired recovery from EAE [20]. Although the reason for this discrepancy is not clear at present, the difference between the two mutant strains may arise from the difference in the targeting strategies for the *Ptprz-*gene disruption or in the genetic background. Their knockout strain was generated by the replacement of an exon encoding a portion of the extracellular CAH domain with a *pgk-neo* cassette [19], which remains a theoretical concern for unexpected expression of an aberrant N-terminal fragment of Ptprz. In addition, the mixed genetic-background of 129Svev x Swiss Webster mice might cause phenotypic variation due to genetic heterogeneity in the EAE experiments [20]. In this regard, our *Ptprz*-deficient mice are generated by an in frame insertion of *LacZ* just after the translational initiation codon in exon 1 of the *Ptprz* gene, in which the marker protein β-galactosidase is expressed instead of Ptprz under the control of the *Ptprz* gene regulatory unit [8], and we used mice obtained by backcrossing for more than ten generations to the inbred C57BL/6 strain in the present study.

## Materials and Methods

### Antibodies

The purified rabbit polyclonal antibody against phosphorylated Tyr 1105 of p190RhoGAP (anti-pY1105) was described previously [15]. Anti-Ptprz-S is a rabbit polyclonal antibody against the extracellular region of Ptprz [21]. We used commercially available antibodies against phosphotyrosine (PY20, GE Healthcare), MBP (cat no. sc-13914; Santa Cruz), p190RhoGAP (cat no. 610150; BD Biosciences), the intracellular region of Ptprz (anti-RPTPβ, cat no. 610180; BD Biosciences), Fyn (cat no. P2992; Sigma-Aldrich), phosphorylated Tyr 420 of Fyn (cat no. 2101; Cell signaling), phosphorylated Tyr 531 of Fyn (cat no. 2105; Cell signaling), Iba1 (cat no. 019-19741; Wako Pure Chemical), CD3 (cat no. ab5690; abcam), Olig2 (cat no. AF2418; R&D systems), and NG2 chondroitin sulfate proteoglycan (cat no AB5320; Millipore).

### Mice

Wild-type and *Ptprz*-deficient mice [8] backcrossed with the inbred C57BL/6 strain for more than ten generations were used. Mice were given free access to food and water at constant temperature (23°C) and humidity (50%) under a 12-hour light-dark cycle. All animal experiments were performed according to the guidelines of Animal Care with approval by the Committee for Animal Research, National Institutes of Natural Sciences.

### Primary culture and immunofluorescence cell staining

The primary culture of mouse oligodendrocytes was performed as described previously [44] with some modifications. Briefly, whole brains (from mouse pups at postnatal day 1) were dissociated using a papain dissociation system (Worthington Biochemical). The dissociated cells were plated on dishes coated with 50 µg/ml of poly-L-ornithine, and cultured in Dulbecco's modified Eagle's medium mixed 1∶1 with Ham's F-12 (DMEM/F12, Invitrogen) supplemented with 0.5% fetal calf serum, 100 µg/ml of bovine serum albumin, N2 supplement (Invitrogen), 10 µg/ml of the AA homodimeric form of platelet-derived growth factor (PDGF-AA, Wako Pure Chemical), 10 nM biotin (Sigma-Aldrich) and 30 ng/ml of thyronine/thyroxine (Sigma-Aldrich) in a humidified atmosphere of 8.5% CO_2_ in air at 37°C.

### Immunoprecipitation and Western blotting

For the Western blotting, mouse tissues (the cerebral cortex and the third to sixth lumbar vertebrae) removed quickly were extracted with a lysis buffer [100 mg wet tissue/500 µl of lysis buffer: 1% NP-40 in 10 mM Tris-HCl, pH 7.4, 150 mM NaCl (TBS) containing 1 mM vanadate and protease inhibitors (EDTA-free complete, Roche Molecular Biochemicals)] on ice, and the supernatant was collected by centrifugation at 10,000× *g* for 15 min. To analyze the phosphorylation of p190RhoGAP or Fyn, immunoprecipitation was performed as follows: the lumbar spinal cord extract (300 µl; adjusted to 8 mg protein/ml for p190RhoGAP immunoprecipitation, or 1 mg protein/ml for Fyn) was preincubated with 1 µg of anti-p190RhoGAP, or anti-Fyn antibody for 2 h. The immunocomplexes were precipitated using 25 µl of Protein G Sepharose 4FF (GE Healthcare), washed with the lysis buffer. The bound proteins were subjected to SDS-PAGE followed by Western blotting with respective antibodies. The detection of the antibody reactions was performed with an ECL Western blotting system (GE Healthcare).

### Immunohistochemistry and TUNEL assay

The fifth lumbar spinal cords were dissected from mice, fixed with 4% paraformaldehyde in 4.3 mM Na_2_HPO_4_, 1.4 mM KH_2_PO_4_, 137 mM NaCl, and 2.7 mM KCl, pH 7.3 (PBS), and then embedded in paraffin. Immunohistochemistry was performed as described previously [45]. Briefly, deparaffinized sections (7 µm) were microwaved in 10 mM citrate buffer, pH 6.0 for 15 min, and pretreated with 3% hydrogen peroxide in TBS containing 0.04% NP-40. After blocking with 4% nonfat dry milk and 0.1% Triton X-100 in TBS, the sections were incubated with respective antibodies overnight at 4°C and the binding of specific antibodies was detected with DAKO Envision System (DAKO) or Alexa-conjugated secondary antibodies (Invitrogen). The TUNEL assay was applied to formalin-fixed and paraffin-embedded tissues by using an *in situ* Apoptosis Detection Kit (Takara-Bio) according to the manufacturer's instructions.

### Electron microscopy and morphometry

Mice were anesthetized and perfused with 4% paraformaldehyde and 1% glutaraldehyde in 0.1 M cacodylate buffer, pH7.4. A 1-mm-square block of the corpus callosum was quickly removed from the brain, fixed with 2% paraformaldehyde and 2% glutaraldehyde, and postfixed with 2% osmium tetroxide for 2 h at 4°C. The fixed specimens were dehydrated with a graded series of ethanol solutions, and embedded in resin Quetol 812 (Nisshin EM). Ultrathin sections (80 nm) were prepared from Epon-embedded tissues, stained with uranyl acetate and a lead stain solution (Sigma-Aldrich), and then analyzed with a JEM-1200EX electron microscope (Jeol).

### Induction of EAE and grading of disabilities

Mice (female, 2 months old) were subcutaneously immunized with 200 µg of myelin/oligodendrocyte glycoprotein (MOG) peptide (amino acids 35–55) emulsified with incomplete Freund's adjuvant containing 4 mg/ml of Mycobacterium tuberculosis (Day 0), followed by an interperitoneal administration of 400 ng of pertussis toxoid on Day 0 and Day 2. The animals were examined for disabilities until Day 50 with clinical grading as follows: 0, no signs; 1, limp tail; 2, ataxia and/or paresis of hindlimbs; 3, paralysis of hindlimbs and/or paresis of forelimbs; 4, tetraparalysis; 5, moribund or death. At the time of sacrifice, the mice anesthetized were perfused transcardially with 4% paraformaldehyde in PBS. The fifth lumbar vertebra was post-fixed overnight in the same fixative solution, and then embedded in paraffin. Deparaffinized sections (7 µm) were stained with hematoxylin and eosin, Klüver-Barrera, and Bielschowsky silver impregnation to detect inflammatory infiltrates, demyelination and axonal loss, respectively.

### T-cell proliferation assay

T-cell proliferation was measured using a Cell Proliferation ELISA BrdU (colorimetric) Kit (Roche Applied Science) according to the manufacturer's instructions. Briefly, T-cells were isolated from the axillary and inguinal lymph nodes of mice 10, 28 and 35 days after immunization with the MOG peptide, or naive mice. The isolated cells were cultivated with RPMI 1640 medium supplemented with 10% fetal calf serum under 5% CO_2_ at 37°C (2×10^4^ cells in 100 µl of medium per well in 96-well plates) in the presence of MOG peptide (1 µg/ml), Dynabeads with Mouse T-Activator CD3/CD28 (2×10^4^ beads per well, Invitrogen), or vehicle (PBS). The BrdU-labeling reagent was added to cultures 48 h after the stimulations, and incubated for an additional 24 h. The cells were harvested by centrifugation, and the incorporated BrdU was quantified with the peroxidase-labeled antibody to BrdU.

### Statistics and image analyses

Statistical analyses were performed using IBM SPSS Statistics 20 software (International Business Machines). Quantitative image analyses of the tissue section staining or electron microscopy were performed using Image J software (National Institutes of Health), or Adobe Photoshop CS6 software (Adobe Systems).

## Supporting Information

Figure S1 ***A*** **, Schematic representation of Ptprz isoforms and their proteolytic fragments.** Z_A_-ECF or Z_B_-ECF is the extracellular fragment of Ptprz-A or Ptprz-B produced by metalloproteinase-induced shedding, and ZΔE is their counterpart membrane-tethered fragment. Proteolytically released Z_A_-ECF from Ptprz-A has the same structure as Ptprz-S, except their carboxyl termini. Z-ICF is the intracellular fragment cleaved from ZΔE by presenilin/γ-secretase activity [21]. Z-ECFs are extracellular fragments generated by plasmin cleavage [23]. Domains are highlighted in different colors: carbonic anhydrase-like domain (red), fibronectin type III domain (blue), and the PTP-D1 (orange) and PTP-D2 (green) domains. A portion (gray) in Ptprz-A is missing in Ptprz-B. The extracellular region of all three isoforms is modified with chondroitin sulfate (CS) chains. Regions corresponding to the epitopes of antibodies used in this study are indicated by vertical lines. ***B–D***, Ptprz expression in the spinal cord. Western blot analyses of the lumbar spinal cords of 2 months old mice with anti-Ptprz-S (B) and anti-RPTPβ (C). Applied protein amounts were verified by CBB staining (D). Extracts treated with (+) or without (−) chondroitinase ABC (chABC) were analyzed.
(TIF)

Note S1 **All three isoforms of Ptprz are highly glycosylated with chondroitin sulfate (CS) in the adult mouse brain, and therefore the tissue extracts were treated with chABC (Seikagaku) to remove CS chains and resolve their core proteins by SDS-PAGE** [**1**] **.** As shown in Figure S1B, a total of six bands were detected in the extracts of the spinal cord with anti-Ptprz-S. The 300-kDa and 250-kDa species represent the core proteins of Ptprz-S (or Z_A_-ECF) and Ptprz-B, respectively, and the other four lower molecular-size species are the proteolytic fragments of Ptprz isoforms produced by extracellular cleavages in vivo by several proteases including metalloproteinases [21] or plasmin [23] (see Figure S1A). When the same blot was reprobed with anti-RPTPβ which recognizes the intracellular region, the full-length Ptprz-B (250-kDa) was deteted together with several proteolytic C-terminal fragments including ZΔE/Z-ICF (Figure S1C). All of these immunoreactive bands were absent from the spinal cord of Ptprz-deficient mice, verifying the specificity of the two antibodies.
(DOC)

Figure S2 **RT-PCR analyses of Ptprz expression.** ***A***, The fifth lumbar spinal cord. ***B***, T-cells taken from the axillary and inguinal lymph nodes. RT-PCR analyses were performed as were performed as described previously [45] with slight modifications. Briefly, total RNA was isolated with a TRIzol Reagent kit (Invitrogen) and cDNA was synthesized using the SuperScript III Reverse Transcriptase kit (Invitrogen) with random hexamer primers. Specific cDNA regions for the three isoforms of Ptprz or glyceraldehyde-3-phosphate dehydrogenase (GAPDH, used as a control) were then amplified by PCR with the following primer sets: *Ptprz-A*, forward 5′-caggagtatccaacagttcagag-3′ and reverse 5′-ttttcagcaagttgtgtgag-3′; *Ptprz-B*, forward 5′-cctccagaccacttgatttg-3′ and reverse 5′-ttttcagcaagttgtgtgag-3′; *Ptprz-S*, forward 5′-aaccagaacgttcaaccatttg-3′ and reverse 5′-tccctacagaaaaggctc-3′; or *GAPDH*, forward 5′-ggatttggccgtattgggcgcctggtcacc-3′ and reverse 5′-tcttctgggtggcagtgatggcatggactg-3′.
(TIF)

Figure S3 **Reduced histological severity of EAE in** ***Ptprz*** **-deficient mice.** Klüver-Barrera (***A***) and Bielschowsky silver staining (***B***) of the spinal cord obtained from wild-type and *Ptprz*-deficient mice 50 days after MOG immunization. The lower images are enlargements of the areas enclosed by squares in the upper images. Scale bars, 500 µm. The extent of demyelination and axon injury was determined by Klüver-Barrera staining and Bielschowsky silver staining, respectively, and the percentage of damaged areas is shown at the right of each panel. Data are the mean ± SEM (*Ptprz* ^+/+^, *n* = 14; *Ptprz* ^−/−^, *n* = 12). *p<0.05 and **p<0.01 (Student's *t*-test).
(TIF)

Figure S4 **Characterization of TUNEL-positive apoptotic cells in the spinal cord after EAE induction.** ***A***, Immunohistchemistry of the spinal cord. After the TUNEL staining of representative spinal cord sections obtained from wild-type and *Ptprz*-deficient mice 35 days after MOG immunization, the sections were then stained with anti-Olig2 (for oligodendrocyte lineage cells), anti-CD3 (for T-cells), or anti-Iba1 (for macrophages/microglia). Scale bars, 50 µm. ***B***, The percentages of double positive cells (Olig2/TUNEL, CD3/TUNEL, or Iba1/TUNEL) among the TUNEL-positive cells. Data are the mean ± SEM (*n* = 4 for each group). No significant differences were detected between the two genotypes.
(TIF)

Figure S5 **No genotypic differences in infiltrating T-cells and macrophages/microglia within the spinal cord after EAE induction.** ***A***, Hematoxylin and eosin staining of the spinal cord 50 days after MOG immunization. The lower images are enlargements of the areas enclosed by squares in the upper images. Scale bars, 500 µm. The numbers of infiltrating cells per section are shown at the right. Data are the mean ± SEM (*Ptprz* ^+/+^, *n* = 14; *Ptprz* ^−/−^, *n* = 12). ***B***, Immunohistochemistry of infiltrating T-cells (detected with anti-CD3) or macrophages/microglia (with anti-Iba1) in the spinal cords obtained from wild-type and *Ptprz*-deficient mice 50 days after MOG immunization. Scale bars, 50 µm. The numbers of CD3-positive or Iba1-positive cells are shown at the right. Data are the mean ± SEM (*n* = 6 for each group). No significant differences were detected between the two genotypes.
(TIF)

Figure S6 **Reduced MBP loss in** ***Ptprz*** **-deficient mice after EAE induction.** Anti-MBP staining of the spinal cord sections from wild-type and *Ptprz*-deficient mice. Data at 10 (***A***) or 28 (***B***) days after the MOG immunization are shown together with data for non-immunized control mice. Lower images are enlargements of the areas enclosed by squares in the upper images. Scale bars, 500 µm. The densitometric data for MBP signals were expressed as the relative change (fold-increase) compared with the data for non-immunized wild-type mice, and shown at the right of each panel. Data are the mean ± SEM (*n* = 9 for each group). **p<0.01 (Student's *t*-test). a.u., arbitrary unit.
(TIF)
