# Supplementary figures and images for: Protein Tyrosine Phosphatase Receptor Type Z Negatively Regulates Oligodendrocyte Differentiation and Myelination
Source: PLoS One. 2012 Nov 7;7(11):e48797. doi: 10.1371/journal.pone.0048797 (PMC3492236; doi:10.1371/journal.pone.0048797)

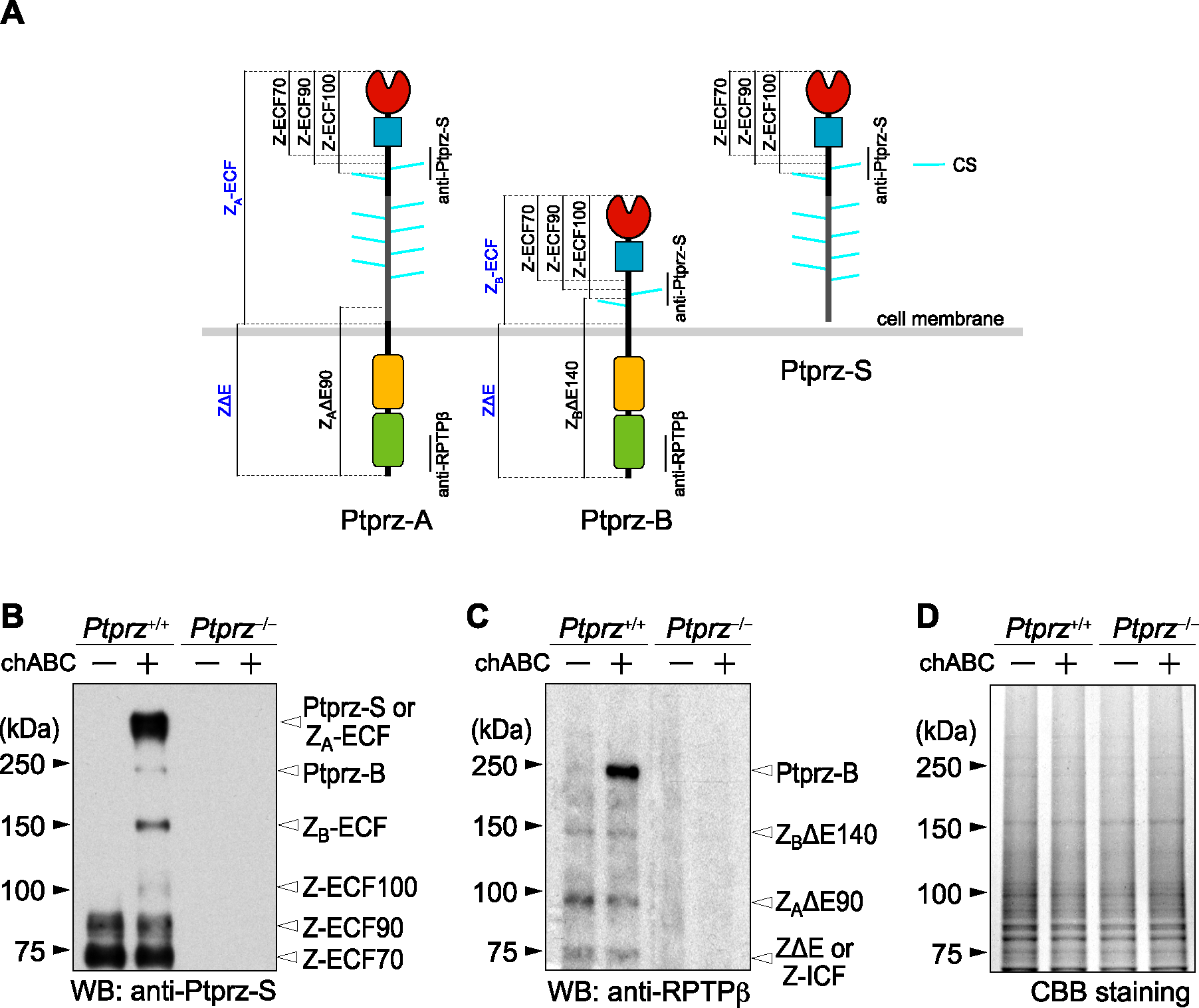

Supplement: Figure S1 — A , Schematic representation of Ptprz isoforms and their proteolytic fragments. ZA-ECF or ZB-ECF is the extracellular fragment of Ptprz-A or Ptprz-B produced by metalloproteinase-induced shedding, and ZΔE is their counterpart membrane-tethered fragment. Proteolytically released ZA-ECF from Ptprz-A has the same structure as Ptprz-S, except their carboxyl termini. Z-ICF is the intracellular fragment cleaved from ZΔE by presenilin/γ-secretase activity [21]. Z-ECFs are extracellular fragments generated by plasmin cleavage [23]. Domains are highlighted in different colors: carbonic anhydrase-like domain (red), fibronectin type III domain (blue), and the PTP-D1 (orange) and PTP-D2 (green) domains. A portion (gray) in Ptprz-A is missing in Ptprz-B. The extracellular region of all three isoforms is modified with chondroitin sulfate (CS) chains. Regions corresponding to the epitopes of antibodies used in this study are indicated by vertical lines. B–D, Ptprz expression in the spinal cord. Western blot analyses of the lumbar spinal cords of 2 months old mice with anti-Ptprz-S (B) and anti-RPTPβ (C). Applied protein amounts were verified by CBB staining (D). Extracts treated with (+) or without (−) chondroitinase ABC (chABC) were analyzed. (TIF) [file pone.0048797.s001.tif]

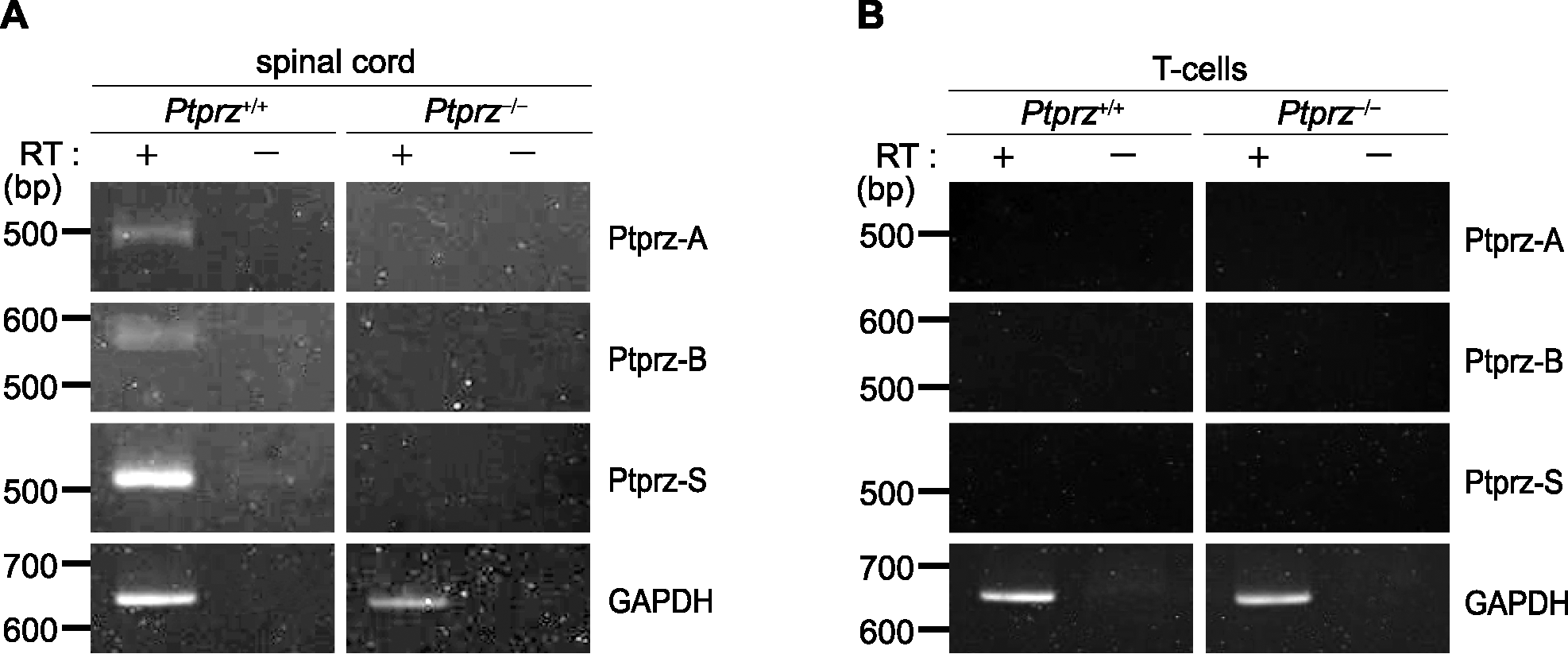

Supplement: Figure S2 — RT-PCR analyses of Ptprz expression. A, The fifth lumbar spinal cord. B, T-cells taken from the axillary and inguinal lymph nodes. RT-PCR analyses were performed as were performed as described previously [45] with slight modifications. Briefly, total RNA was isolated with a TRIzol Reagent kit (Invitrogen) and cDNA was synthesized using the SuperScript III Reverse Transcriptase kit (Invitrogen) with random hexamer primers. Specific cDNA regions for the three isoforms of Ptprz or glyceraldehyde-3-phosphate dehydrogenase (GAPDH, used as a control) were then amplified by PCR with the following primer sets: Ptprz-A, forward 5′-caggagtatccaacagttcagag-3′ and reverse 5′-ttttcagcaagttgtgtgag-3′; Ptprz-B, forward 5′-cctccagaccacttgatttg-3′ and reverse 5′-ttttcagcaagttgtgtgag-3′; Ptprz-S, forward 5′-aaccagaacgttcaaccatttg-3′ and reverse 5′-tccctacagaaaaggctc-3′; or GAPDH, forward 5′-ggatttggccgtattgggcgcctggtcacc-3′ and reverse 5′-tcttctgggtggcagtgatggcatggactg-3′. (TIF) [file pone.0048797.s003.tif]

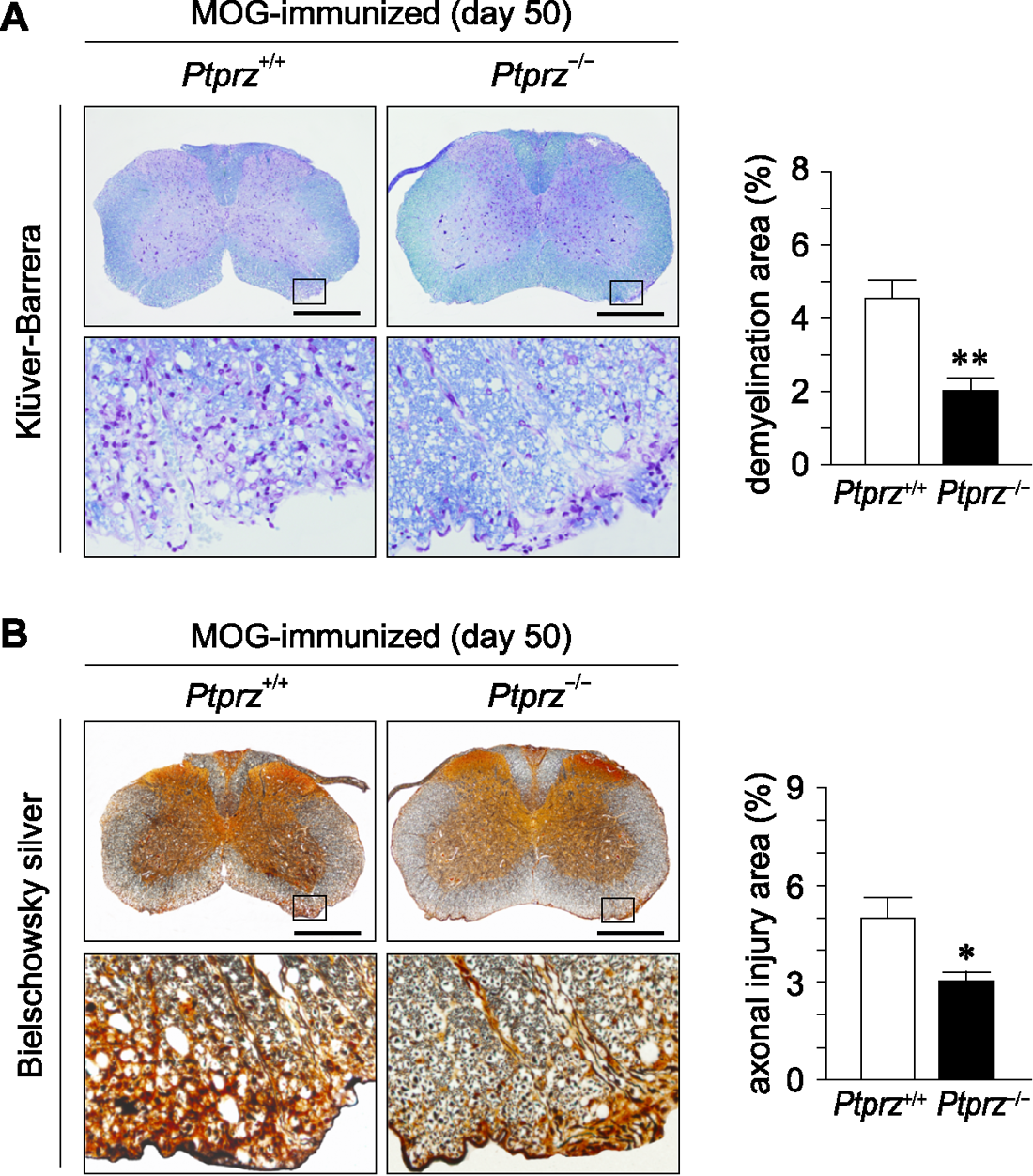

Supplement: Figure S3 — Reduced histological severity of EAE in Ptprz -deficient mice. Klüver-Barrera (A) and Bielschowsky silver staining (B) of the spinal cord obtained from wild-type and Ptprz-deficient mice 50 days after MOG immunization. The lower images are enlargements of the areas enclosed by squares in the upper images. Scale bars, 500 µm. The extent of demyelination and axon injury was determined by Klüver-Barrera staining and Bielschowsky silver staining, respectively, and the percentage of damaged areas is shown at the right of each panel. Data are the mean ± SEM (Ptprz +/+, n = 14; Ptprz −/−, n = 12). *p<0.05 and **p<0.01 (Student's t-test). (TIF) [file pone.0048797.s004.tif]

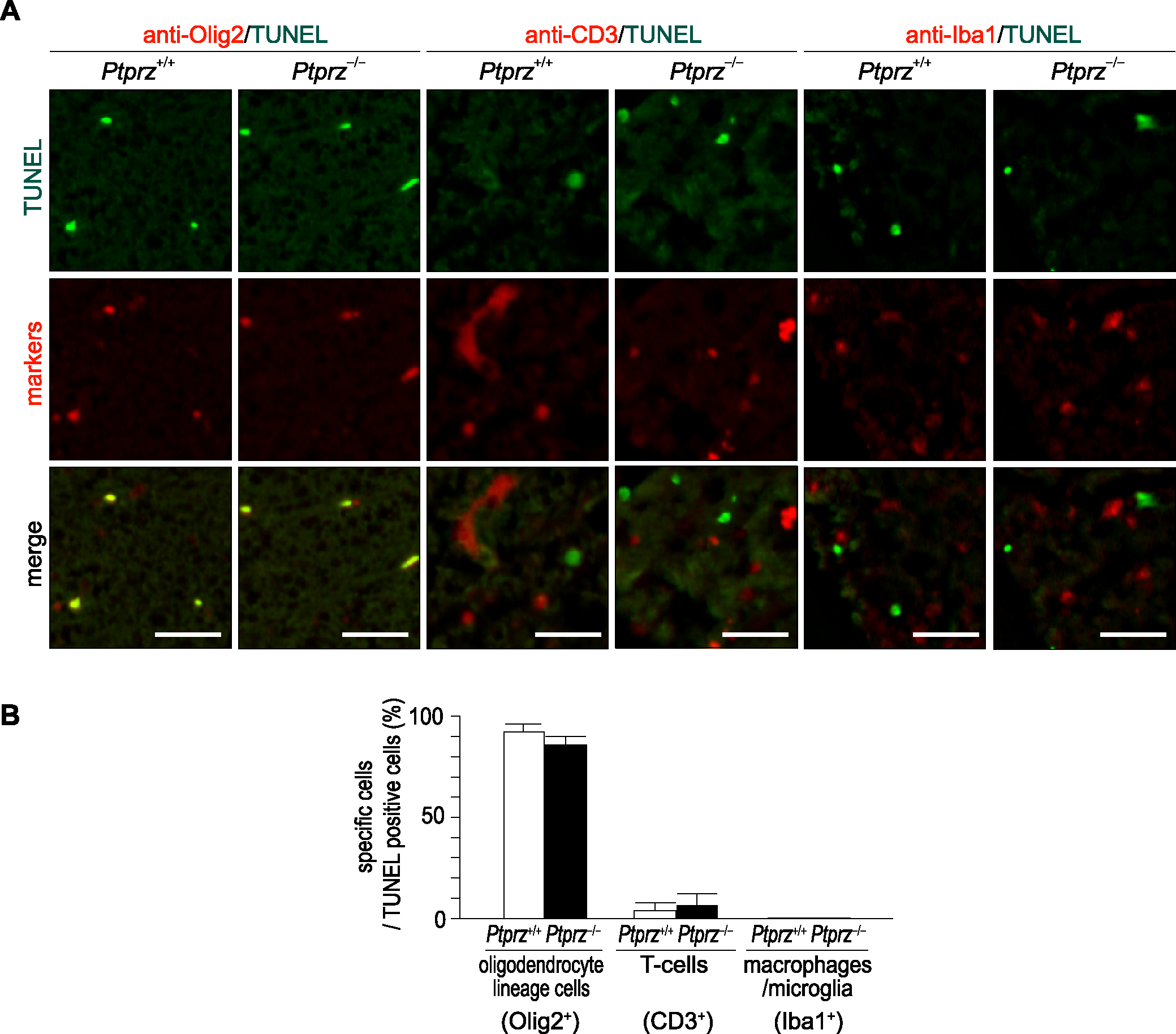

Supplement: Figure S4 — Characterization of TUNEL-positive apoptotic cells in the spinal cord after EAE induction. A, Immunohistchemistry of the spinal cord. After the TUNEL staining of representative spinal cord sections obtained from wild-type and Ptprz-deficient mice 35 days after MOG immunization, the sections were then stained with anti-Olig2 (for oligodendrocyte lineage cells), anti-CD3 (for T-cells), or anti-Iba1 (for macrophages/microglia). Scale bars, 50 µm. B, The percentages of double positive cells (Olig2/TUNEL, CD3/TUNEL, or Iba1/TUNEL) among the TUNEL-positive cells. Data are the mean ± SEM (n = 4 for each group). No significant differences were detected between the two genotypes. (TIF) [file pone.0048797.s005.tif]

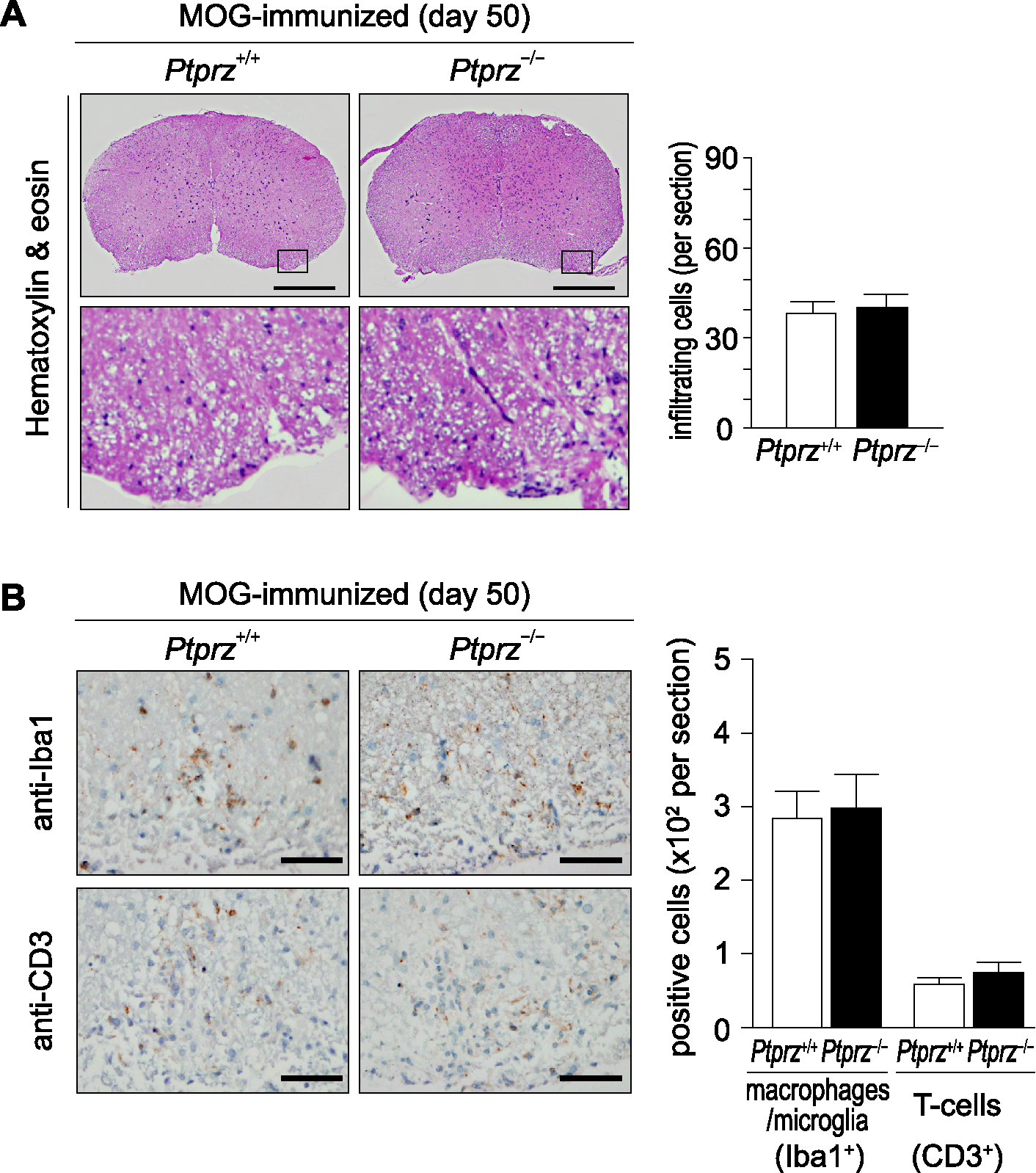

Supplement: Figure S5 — No genotypic differences in infiltrating T-cells and macrophages/microglia within the spinal cord after EAE induction. A, Hematoxylin and eosin staining of the spinal cord 50 days after MOG immunization. The lower images are enlargements of the areas enclosed by squares in the upper images. Scale bars, 500 µm. The numbers of infiltrating cells per section are shown at the right. Data are the mean ± SEM (Ptprz +/+, n = 14; Ptprz −/−, n = 12). B, Immunohistochemistry of infiltrating T-cells (detected with anti-CD3) or macrophages/microglia (with anti-Iba1) in the spinal cords obtained from wild-type and Ptprz-deficient mice 50 days after MOG immunization. Scale bars, 50 µm. The numbers of CD3-positive or Iba1-positive cells are shown at the right. Data are the mean ± SEM (n = 6 for each group). No significant differences were detected between the two genotypes. (TIF) [file pone.0048797.s006.tif]

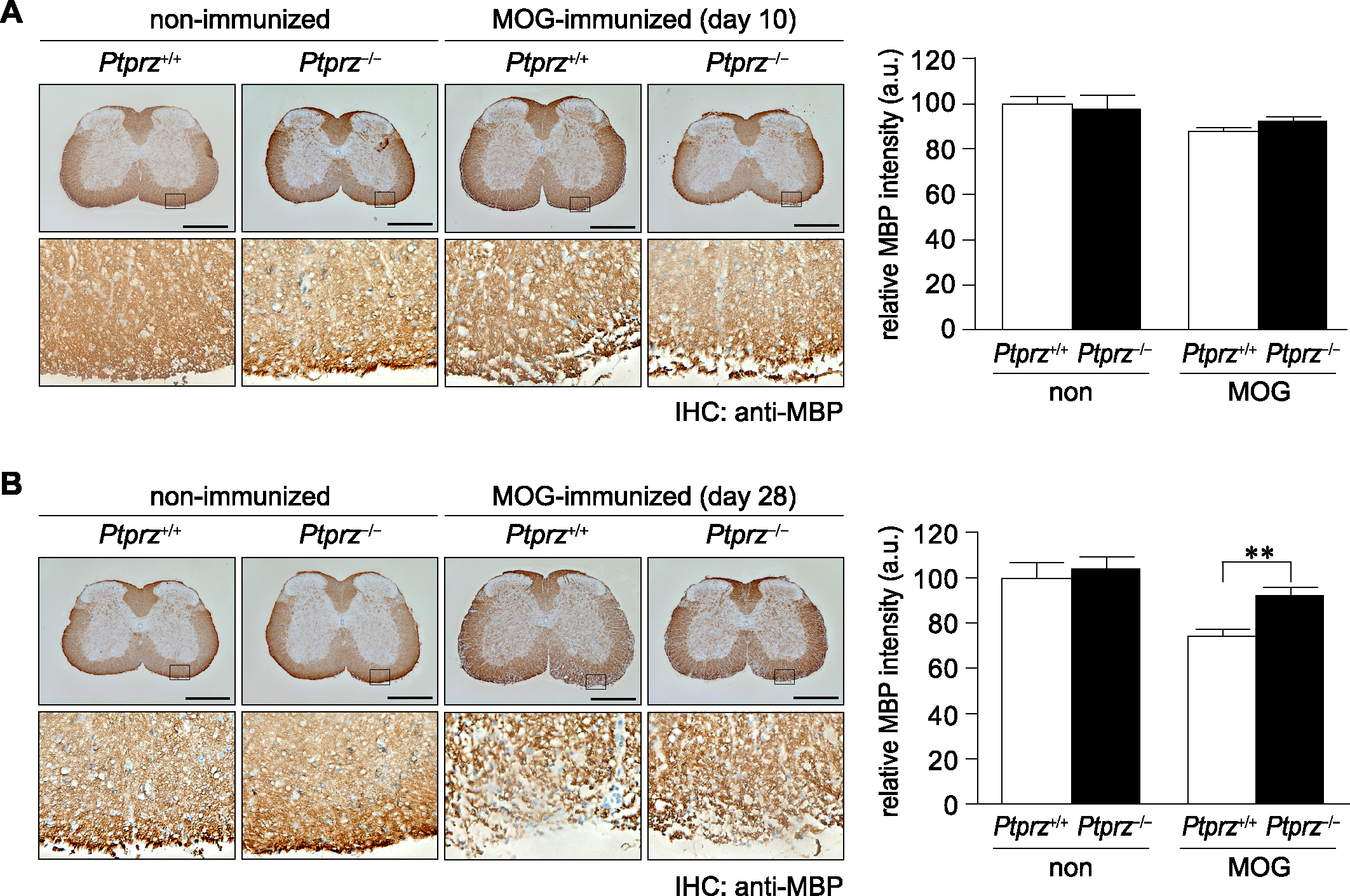

Supplement: Figure S6 — Reduced MBP loss in Ptprz -deficient mice after EAE induction. Anti-MBP staining of the spinal cord sections from wild-type and Ptprz-deficient mice. Data at 10 (A) or 28 (B) days after the MOG immunization are shown together with data for non-immunized control mice. Lower images are enlargements of the areas enclosed by squares in the upper images. Scale bars, 500 µm. The densitometric data for MBP signals were expressed as the relative change (fold-increase) compared with the data for non-immunized wild-type mice, and shown at the right of each panel. Data are the mean ± SEM (n = 9 for each group). **p<0.01 (Student's t-test). a.u., arbitrary unit. (TIF) [file pone.0048797.s007.tif]
