# Supplementary material for: Protein Tyrosine Phosphatase Receptor Type Z Negatively Regulates Oligodendrocyte Differentiation and Myelination
Source: PLoS One. 2012 Nov 7;7(11):e48797. doi: 10.1371/journal.pone.0048797 (PMC3492236; doi:10.1371/journal.pone.0048797)
Supplement: Note S1 — All three isoforms of Ptprz are highly glycosylated with chondroitin sulfate (CS) in the adult mouse brain, and therefore the tissue extracts were treated with chABC (Seikagaku) to remove CS chains and resolve their core proteins by SDS-PAGE [1] . As shown in Figure S1B, a total of six bands were detected in the extracts of the spinal cord with anti-Ptprz-S. The 300-kDa and 250-kDa species represent the core proteins of Ptprz-S (or ZA-ECF) and Ptprz-B, respectively, and the other four lower molecular-size species are the proteolytic fragments of Ptprz isoforms produced by extracellular cleavages in vivo by several proteases including metalloproteinases [21] or plasmin [23] (see Figure S1A). When the same blot was reprobed with anti-RPTPβ which recognizes the intracellular region, the full-length Ptprz-B (250-kDa) was deteted together with several proteolytic C-terminal fragments including ZΔE/Z-ICF (Figure S1C). All of these immunoreactive bands were absent from the spinal cord of Ptprz-deficient mice, verifying the specificity of the two antibodies. (DOC) [file pone.0048797.s002.doc]

**Supplementary Note**

### All three isoforms of Ptprz are highly glycosylated with chondroitin sulfate (CS) in the adult mouse brain, and therefore the tissue extracts were treated with chABC (Seikagaku) to remove CS chains and resolve their core proteins by SDS-PAGE [1]. As shown in Supplementary Figure S1B, a total of six bands were detected in the extracts of the spinal cord with anti-Ptprz-S. The 300-kDa and 250-kDa species represent the core proteins of Ptprz-S (or Z_A_-ECF) and Ptprz-B, respectively, and the other four lower molecular-size species are the proteolytic fragments of Ptprz isoforms produced by extracellular cleavages in vivo by several proteases including metalloproteinases [1] or plasmin [2] (see Supplementary Figure S1A). When the same blot was reprobed with anti-RPTPβ which recognizes the intracellular region, the full-length Ptprz-B (250-kDa) was deteted together with several proteolytic C-terminal fragments including ZΔE/Z-ICF (Supplementary Figure S1C). All of these immunoreactive bands were absent from the spinal cord of Ptprz-deficient mice, verifying the specificity of the two antibodies.

### References

1. Chow JP, Fujikawa A, Shimizu H, Suzuki R, Noda M (2008) Metalloproteinase- and γ-secretase-mediated cleavage of protein-tyrosine phosphatase receptor type Z. J Biol Chem 283: 30879-30889.
2. Chow JP, Fujikawa A, Shimizu H, Noda M (2008) Plasmin-mediated processing of protein tyrosine phosphatase receptor type Z in the mouse brain. Neurosci Lett 442: 208-212.
3. Fujikawa A, Shirasaka D, Yamamoto S, Ota H, Yahiro K, et al. (2003) Mice deficient in protein tyrosine phosphatase receptor type Z are resistant to gastric ulcer induction by VacA of *Helicobacter pylori*. Nat Genet 33: 375-381.
